# Preoperative arterial and venous CT radiomics for survival prediction after pylorus preserving pancreatoduodenectomy in pancreatic head cancer

**DOI:** 10.1038/s41598-025-09969-x

**Published:** 2025-07-10

**Authors:** Annika Rastkhiz, Ali Rastkhiz, Felicia Erb, Maximilian Schulze-Hagen, Florian Tom Ulmer, Ulf Peter Neumann, Gregory van der Kroft, Christiane Katharina Kuhl, Daniel Truhn, Teresa Lemainque

**Affiliations:** 1 RWTH Aachen University, Aachen, Germany; 2 Städtisches Klinikum Solingen, Solingen, Germany; 3 Universitätsklinikum Essen, Essen, Germany; 4 Erasmus University Medical Center, Rotterdam, Netherlands; 5 Uniklinik RWTH Aachen, Aachen, Germany

**Keywords:** Pancreatic cancer, Radiomics, Survival prediction, CT imaging, Pancreatoduodenectomy, Gastroenterology, Medical research, Oncology

## Abstract

Pancreatic cancer (PaCa) is the seventh leading cause of cancer deaths globally, with limited detection and treatment. Pancreatoduodenectomy (PD) is the primary surgical intervention for resectable pancreatic head cancers (PaHCa), but its complexity necessitates prognostic tools. This study evaluates the arterial and venous phase radiomic features role from preoperative CT scans in predicting survival for PaHCa patients undergoing pylorus-preserving pancreatoduodenectomy (PPPD). A retrospective analysis was conducted on 42 PaHCa patients (mean age 63.3 ± 10 years; 20 males, 22 females) who underwent PPPD between 2010 and 2017. Radiomic features were extracted from arterial and venous phase CT images, and a gradient boosting survival model was applied for survival prediction using cross-validation. Ethical approval (Approval number: EK028/19, date: 03.05.2019) was granted, and informed consent was waived due to the retrospective nature of the study and all experiments were performed in accordance with relevant guidelines and regulations. No identifying information or images are included in this manuscript. Survival analysis revealed no significant differences when using arterial (*p* = 0.161) or venous (*p* = 0.668) phase features alone. However, combining arterial and venous phase features significantly improved survival prediction (*p* = 0.007). Key predictive features included “Shape: Sphericity” and “Gray Level Size-Zone Non-Uniformity (GLSZM)”. Combining arterial and venous phase radiomic features enhances survival prediction in PPPD-treated PaHCa patients, highlighting the potential of multi-phase CT radiomics for personalized treatment strategies. Radiomics-based survival prediction of PaHCa prior to patients undergoing PPPD may guide clinical decision-making and improve personalized treatment planning.

## Introduction

Pancreatic cancer (PaCa), particularly pancreatic head cancer (PaHCa), is the 12th most prevalent cancer worldwide and the seventh leading cause of cancer-related deaths, as reported by the International Agency for Research on Cancer in 2020^1^. The mortality rate for PaCa is expected to increase by 25% by 2025, making it the third most common cause of cancer-related deaths, after lung and colorectal cancers^2^. The main risk factors for PaCa include type 2 diabetes, obesity, smoking, and family history. Unfortunately, the majority of patients present at advanced stages due to the lack of symptoms when the tumor is localized^3^. In addition, early diagnosis of PaCa is challenging, as few solid biomarkers and liquid biopsy techniques are available^4^. While 60–70% of PaCas occur in the head of the pancreas, 20–25% are located in the body or tail^5^, and the uncinate process accounts for 3–11% of all pancreatic ductal adenocarcinoma (PDAC) cases^6^. To present, no biomarker has been validated for early identification of PaHCa^7^. Surgical resection remains the primary treatment for PaHCa, but the prognosis is generally poor^8^.

Pancreatoduodenectomy (PD) is the standard surgical intervention for resectable or borderline resectable PaHCa. Borderline resectable tumors often require advanced techniques, such as vascular resection and reconstruction, which have expanded the pool of eligible patients but may also increase complication rates^9^. These challenges underscore the need for reliable prognostic tools to guide treatment decisions and improve patient outcomes.

Radiomics is based on the idea that biomedical imaging contains information that indicates disease-specific processes and is obtainable through quantitative image analysis^10^. It includes several metric extractions from images, which convey information regarding the cancer environmental conditions and tumor phenotype^11^. In PaCa, deep learning and radiomics-based systems have shown promise in detecting lesions, staging, and survival prediction^12^. Several studies exist that investigated the potential of computed tomography (CT)-based radiomics for survival prediction after pylorus-preserving pancreaticoduodectomy (PPPD); however, these studies extracted the radiomic features from pre-operative CT images acquired in the venous phase^13–17^. To the best of our knowledge, the influence of the contrast phase on survival prediction remains yet to be elucidated.

This study aims to evaluate the influence of contrast phase on survival prediction in PaHCa patients undergoing PPPD. We hypothesize that combining radiomic features from both arterial and venous phase CT scans will improve survival prediction accuracy compared to using either phase alone. By addressing this gap, our work seeks to advance the application of radiomics in personalized treatment planning for PaHCa patients.

## Materials and methods

### Study design

**Fig. 1:**
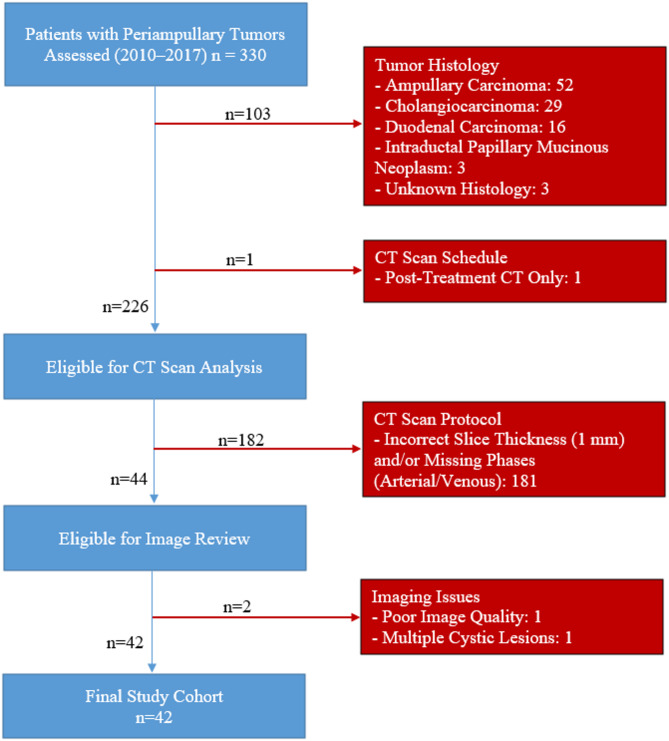
Flowchart of patient inclusion and exclusion criteria. Exclusion criteria are displayed on the right in red boxes; patients included in the final analysis are shown in blue.

This study is a retrospective case-control study approved by the local ethical committee (Approval number: EK028/19, date: 03.05.2019). Due to the retrospective nature of the study, informed consent was waived. All methods were performed in accordance with relevant institutional guidelines and regulations. No identifying information or images are included in this manuscript. Patients who presented between 2010 and 2017 in the RWTH Aachen University Hospital with a diagnosis of PaHCa, who had had a PPPD performed as part of their treatment including a record of preoperative CT scans in arterial and venous phase at a slice thickness of 1 mm, and who had a postoperative follow-up of at least 30 days, were eligible for inclusion into the study. Figure 1 provides a visual summary of the patient inclusion and exclusion criteria used in this study.

Prior to surgery, the patients’ overall health was assessed by the American Society of Anesthesiologists (ASA) score, of which the categories are specified as ASA 1 (normal health), ASA 2 (mild systemic disease), ASA 3 (severe systemic disease), ASA 4 (incapacitating systemic disease), ASA 5 (moribund) and ASA 6 (declared brain-dead organ donor)^18^.

As can be observed from Table 2, the mortality rate showed the most substantial increase between 1 and 3 years after surgery. Also, the median follow-up time was 1,663 days, and the mean follow-up time was approximately 1,555 days (range: 167–2,852 days, as detailed in Table 1).

**Table 1:** Data of the patients with pancreatic head cancer (PaHCa).

Feature of the study cohort		Number	
Gender	Male	20	
	Female	22	
Age (years)	Minimum	36	
	Maximum	83	
	Average ± standard deviation (SD)	63.3 ± 10	
Body mass index (BMI) (kg/m²)	Minimum	18.4	
	Maximum	45.1	
	Median	25.4	
	Interquartile range (IQR)	22.2–30.2	
American Society of Anesthesiologists (ASA)-Score	Minimum	1	2 patients
	Maximum	3	27 patients
	Median	3	
Date of surgery (mm/yyyy)	Earliest date	01/2010	
	Latest date	10/2017	
Follow-up (days)	Minimum	167	
	Maximum	2,852	
	Average	1,555	
	Median	1,663	
Risk group classification	Arterial	High-risk: 22; Low-risk: 20	
	Venous	High-risk: 25; Low-risk: 17	
	Combined	High-risk: 19; Low-risk: 23	

**Table 2:** Survival outcomes and hazard ratios (HR) for high- and low-risk patient groups in arterial, venous, and combined radiomic models.

Metric	Arterial—low risk	Arterial—high risk	Venous—low risk	Venous—high risk	Combined—low risk	Combined—high risk
Median survival (days)	1,693.00	1,558.50	1,564.00	1,675.00	1,743.00	1,472.00
1-Year survival rate	90.0%	95.45%	82.35%	100.0%	86.96%	100.0%
2-Year survival rate	80.0%	72.72%	70.59%	80.0%	78.26%	73.68%
3-Year survival rate	80.0%	59.09%	64.71%	72.0%	73.91%	63.16%
5-Year survival rate	40.0%	40.91%	47.06%	36.0%	47.83%	31.58%
HR	1.20		0.91		1.24	

Median survival (in days), 1- to 5-year survival rates (%), and hazard ratios are presented for each group. Risk classification was determined by the Gradient Boosting Survival Analysis (GBSA) model. Hazard ratios were computed using the Cox proportional hazards model implemented in the lifelines Python package (CoxPHFitter).

Between 2010 and 2017, 330 patients with periampullary tumors underwent examination in RWTH Aachen University Hospital. Of these, 103 patients were excluded due to tumor histology that was not consistent with PaHCa. These included, in particular, ampullary carcinoma (*n* = 52), cholangiocarcinoma (*n* = 29), duodenal carcinoma (*n* = 16), intraductal papillary mucinous neoplasm (*n* = 3), and unknown histology tumors (*n* = 3). Another patient was not included because there was no pre-treatment imaging to go along with post-treatment CT scans.

This left 226 patients to be analyzed by CT scan. However, 182 patients were excluded on the grounds that they had suboptimal imaging protocols that included either a slice thickness > 1 mm or the absence of arterial and/or venous phases. From the remaining 44 patients available for review of images, two were excluded: one due to poor image quality, and another due to the presence of multiple cystic lesions which could not be segmented around.

The final study cohort comprised 42 patients with histologically confirmed PaHCa who underwent PPPD. All included patients had available preoperative contrast-enhanced CT scans in both arterial and venous phases, and complete survival follow-up data. The interval between CT imaging and surgery ranged from 1 to 44 days (median: 6 days; mean: 8.97 days, based on 40 records). As imaging was part of the initial staging process, no patients received neoadjuvant therapy prior to the CT examination.

### Patient cohort and clinical data collection

Clinical and demographic data were collected for all patients included in this study, including sex, age, body mass index (BMI), ASA classification, date of surgery, duration of follow-up, survival time, and postoperative survival at 1 year, 2years, 3 years, and 5 years. This set of variables was used to characterize the study cohort and to explore overall variability in survival. Tables 1 and 2 provide detailed information surrounding this data.

Normality of continuous variables was assessed using the Shapiro-Wilk test. Age was normally distributed (W = 0.9715, *p* = 0.4016), while BMI was not (W = 0.8874, *p* = 0.0008).

### Study endpoints

The primary endpoint was overall survival, defined as the time from the date of surgery to the date of death or last follow-up. Progression-free survival was recorded as a secondary endpoint but not further analyzed in this study.

The median survival time across all models ranged from 1,472 to 1,743 days, depending on the risk group classification (see Table 2). The mean follow-up time for the entire cohort was 1,555 days.

### CT acquisition protocol

Preoperative contrast-enhanced CT scans for tumor staging were performed using three different CT scanner models: the Siemens SOMATOM Definition (software versions: Syngo CT 2010 A and Syngo CT 2006G), TOSHIBA Asteion (software version: V2.05GR001), and PHILIPS Brilliance Big Bore (software versions: 2.3.0 and 3.6.5). Scanning parameters included 120 kVp x-ray tube voltage, 1 mm slice thickness, 314–540 mm FOV and 115–388 mAs exposure, depending on the scanner model and size of the patient.

### Contrast injection protocol

The iodinated contrast agent used for all CT scans was Ultravist-370 (Bayer Vital GmbH, Leverkusen, Germany). 1 mL/kg body weight were administered.

For the arterial and venous phases of imaging, a biphasic protocol of injection was used. The injection rate was 3 mL/sec, but in less-than-optimal peripheral venous catheter situations (e.g., 22 Gauche), this rate was reduced to 2.5 mL/sec. Arterial phase imaging was captured approximately at 30–40 sec post-injection and venous phase imaging was captured at 70–80 sec post-injection. The timing was sustained for optimum enhancement for imaging.

### Segmentation

A.R. (radiology resident with one year of clinical experience) performed the segmentations of the pancreas head using the program ITK-snap (version 3.6.0)^19^. Manual segmentation was performed on the axial slices of the arterial and the venous DICOM CT series. The coronal and sagittal planes were only consulted to fine-tune the segmentations.

A single radiologist independently segmented a subset of 42 cases, resulting in a total of 84 segmentations. A specialist radiologist (M.S.-H., 8 years of experience) subsequently reviewed and validated the segmentations which were adjusted accordingly. Arterial and venous phase segmentations for each patient were performed separately, ensuring consistency upon final evaluation. A time interval of approximately two weeks was maintained between the segmentation of the arterial and venous phases for the same patient. The segmentation process initially focused on axial slices, followed by refinement through coronal and sagittal projections. If discrepancies were identified, all projections and segmentations were iteratively adjusted to ensure optimal accuracy and consistency.

**Fig. 2:**
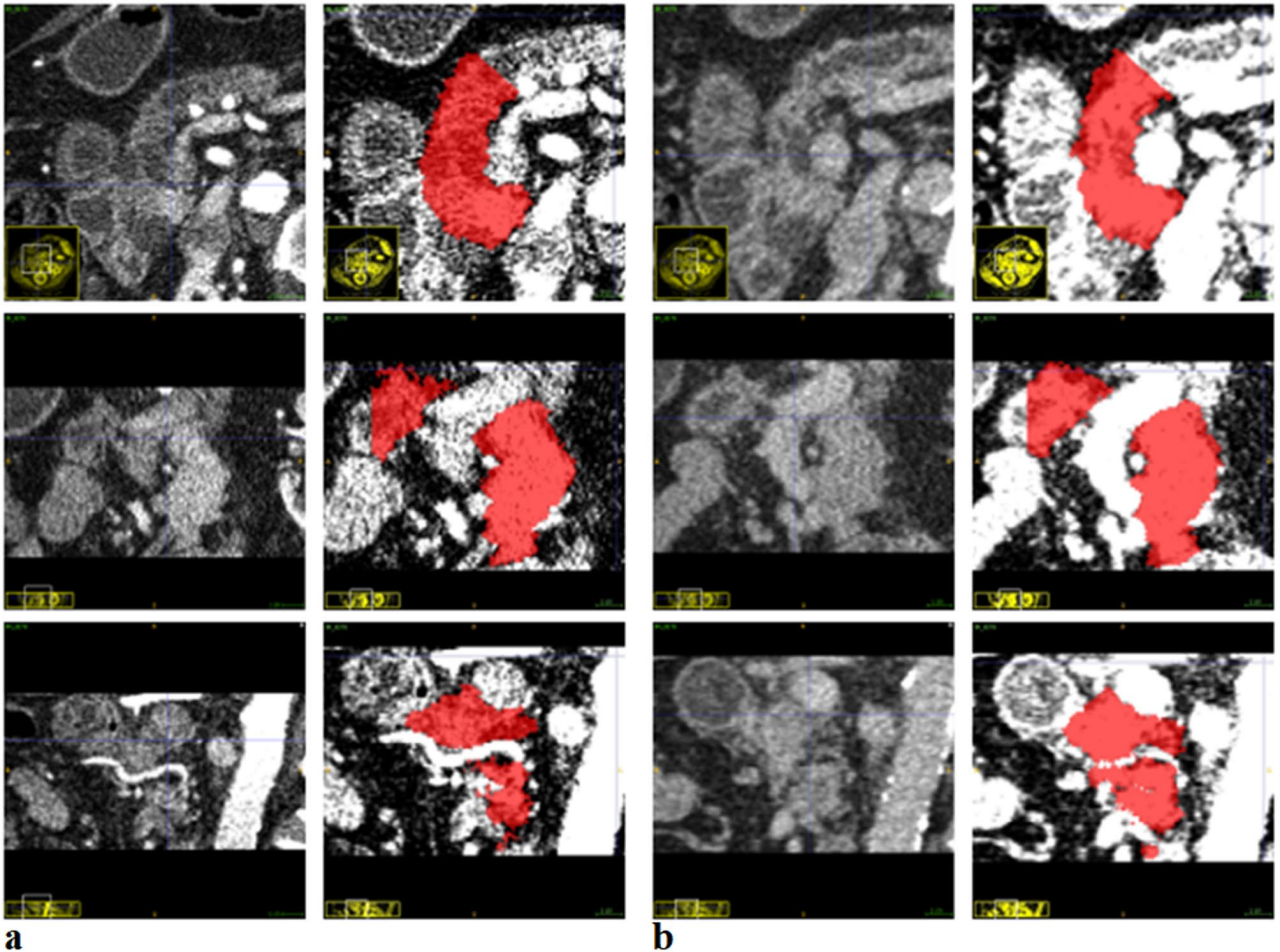
Example segmentation of pancreatic head carcinoma and adjacent pancreatic head parenchyma. (**a**) Arterial phase; (**b**) Venous phase. The axial CT series is presented in the top row, the sagittal series in the middle, and the coronal series at the bottom. The segmentation is drawn in red.

PaHCa along with the adjacent pancreatic head parenchyma was highlighted in red as one region (Fig. 2), up to the point where the arteria mesenterica superior was located closest to the pancreatic parenchyma in the axial CT slices. Without an interface appearing as a reliable intersection to the left, the Processus uncinatus was included completely caudally beneath the duodenum. All 42 venous + arterial segmentation pairs were eligible to be incorporated into the survival analyses. To that purpose, the segmentations and both CT series were stored in Neuroimaging Informatics Technology Initiative (NIFTI) format.

### Radiomic feature extraction

Radiomic feature extraction was done with Python (3.10.13) based on the PyRadiomics library^20^. Radiomics feature extraction was performed with a standardized pipeline to achieve reproducibility and consistency. The DICOM series were converted to NIFTI initially for further processing.

Feature extraction was performed using the PyRadiomics library. Preprocessing of the segmented regions of interest included normalization and resampling. Radiomic features were saved as a CSV file. Although we initially tested a correlation-based feature reduction approach (removing features with > 90% similarity), it led to reduced predictive performance. Therefore, no feature filtering or selection was applied, and all 214 extracted features were retained for model training.

Prior to feature selection, preprocessing steps were applied, including normalization and resampling, to standardize the data and mitigate potential biases.

For both the arterial and the venous CT series and respective segmentations, 107 radiomic features were extracted per patient. The radiomic features were selected from seven groups. Shape features are derived from the approximated shape defined by the triangle mesh. First order statistics features describe the distribution of voxel intensities within the segmented region. Gray level co-occurrence matrix (GLCM) features describe the second-order joint probability function within the segmented region. Gray level dependence matrix (GLDM) features evaluate gray level reliances in an image which relate to the number of linked voxels in a specific distance to the center voxel. In other words, GLDM quantifies the number of times that voxels with the same intensity occur in the direct neighborhood of a voxel. Gray level run length matrix features quantify gray level runs that are defined as the number of consecutive pixels that have the same gray level value. Gray level size-zone non-uniformity (GLSZM) features quantify gray level zones in the segmented region that are defined as a number of connected voxels that share the same gray level intensity. Neighboring gray tone difference matrix features quantify the difference between a gray value and the average gray value of its neighbors within a specific distance^21^.

### Gradient boosting survival analysis with leave-one-out cross validation

The following analyses were performed in Python (version 0.22.0) using the libraries scikit-survival^22^ and scikit-learn^23^. Based on radiomic features, survival time and mortality, a Gradient Boosting Survival Analysis (GBSA) model from scikit-survival was trained. The model coupled the radiomic parameters to the mortality and survival time. Since both arterial and venous radiomic features were available for all 42 patients, three different evaluations were performed. The first evaluation was based on the venous features only, the second evaluation was based on the arterial features only, and the third evaluation was based on the combination of both, i.e., it comprised 214 arterial and venous features in total. These will be referred to as venous analysis, arterial analysis and combined analysis in the following. All previously extracted features were used for model training without any additional feature selection or dimensionality reduction.

To obtain patient-individual risk scores, leave-one-out cross validation was performed^4^. To this purpose, one patient was removed from the dataset at a time and the GBSA model was trained with the remainder of the data. Subsequently, the model response for this patient’s set of radiomic features was queried using the ‘predict’ function of the GBSA model, setting loss=’coxph’. Likewise, this function returns a risk score that can be interpreted as a log Hazard Ratio (HR), similar to the linear predictor of a Cox proportionals model^22,24^. Accordingly, risk scores less than zero were defined as ‘low risk’ and risk scores equal to or larger than zero were defined as ‘high risk’^25^. The process was repeated for every patient. Likewise, a low-risk and a high-risk cohort was obtained for each of the three analyses.

### Feature importance analysis

After training the model, we evaluated feature importance using the built-in attribute from the GBSA model in the scikit-survival Python package. For each of the three models (arterial, venous, and arterial + venous features), we listed the ten most important features. The scores displayed the relative contribution of each feature to the risk prediction from the model. The importance scores are based on the average change in the models’ objective function (log partial likelihood) when the feature is included averaged over all trees in the ensemble. The feature importance scores of a model add up to 1.

### Statistical analysis

In this study, we evaluated the correlation between risk factors and survival using Spearman correlation coefficients. We utilized the interpretation of correlation strength as described by Dancey and Reidy^26^. High-risk and low-risk cohorts were compared by means of Kaplan-Meier survival curves^4^. Survival probabilities were estimated using the Kaplan-Meier estimator implemented in scikit-survival, with 95% confidence intervals (α = 0.05). Three sets of survival curves (arterial, venous, and combined) were generated for high-risk and low-risk patient groups. Differences between survival curves were assessed using the *compare_survival* function with a significance threshold of 0.05.

Survival rates at multiple postoperative time points were calculated based on the number of patients with known survival status at each respective time point, excluding patients with unknown status from the denominator. For example, at the 5th year, survival rates ranged from 31.58 to 47.83% across combined model and risk groups, as shown in Table 2.

## Results

### Patient cohort

Table 1 summarises the patient characteristics. The cohort included 20 male and 22 female patients with a mean age of 63.3 ± 10 years and an age range of 36–83 at the time of surgery. 27 out of 42 patients were classified as ASA 3, i.e., with symptoms of severe systemic disease. Two and 13 patients were classified as ASA 1 and 2, respectively, while no patients were classified as ASA 4–6. The number of days of follow-up was between 167 and 2,852 days, with a median follow-up time of 1,663 days.

Survival curves for the arterial, venous, and combined analyses showed that low-risk patients generally had better survival outcomes than high-risk patients. The combined analysis demonstrated a statistically significant difference between the two risk groups (*p* = 0.007), whereas the arterial and venous analyses did not reach significance (*p* = 0.161 and *p* = 0.668, respectively).

### Weak negative correlations between risk scores and survival for arterial and combined analyses

**Fig. 3:**
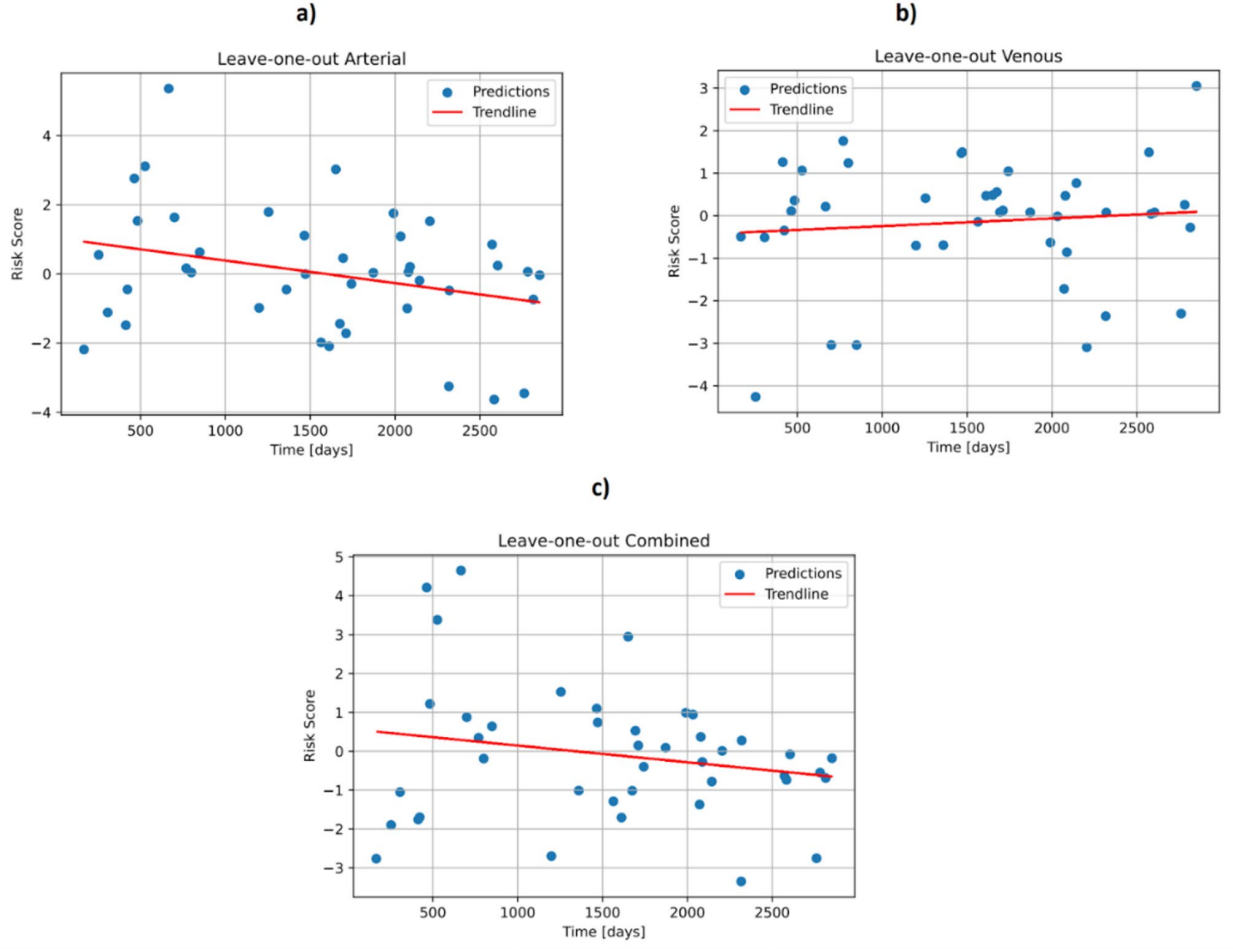
Risk scores of patients after pancreatic surgery calculated based on: (**a**) arterial radiomic features; (**b**) venous radiomic features; and (**c**) combined radiomic features.

Figure 3 indicates the risk scores of each patient versus the survival time after surgery that were obtained from the (a) arterial, (b) venous or (c) combined (i.e., venous and arterial) radiomic features, respectively. Between arterial, venous and combined risk scores, Spearman correlation coefficients of − 0.203, − 0.001 and − 0.09 were calculated, respectively, which indicate weak (and in the venous case, no) correlation^26^. In the arterial analysis, 22 out of 42 patients were classified as high-risk, while 20 out of 42 were low-risk. In the combined analysis, 19 out of 42 patients fell into the high-risk category, and 23 out of 42 were low-risk. For the venous analysis, 25 out of 42 patients were identified as high-risk, whereas 17 out of 42 were in the low-risk group. Of note, the risk score of the venous analysis is, by linear trend, not falling with increased survival time. This is in line with the Spearman correlation coefficient of − 0.001, which indicates no meaningful monotonic relationship between venous risk scores and survival time. In contrast, the risk scores of arterial and combined analyses show a trendline with negative slope and negative correlations as expected. In the case of the venous analysis, the near-zero Spearman correlation coefficient (− 0.001) may reflect a limited sensitivity of venous phase features to perfusion differences, compared to the arterial and combined analyses which showed stronger negative correlations.

### The combined model predicts significantly different survival curves

**Fig. 4:**
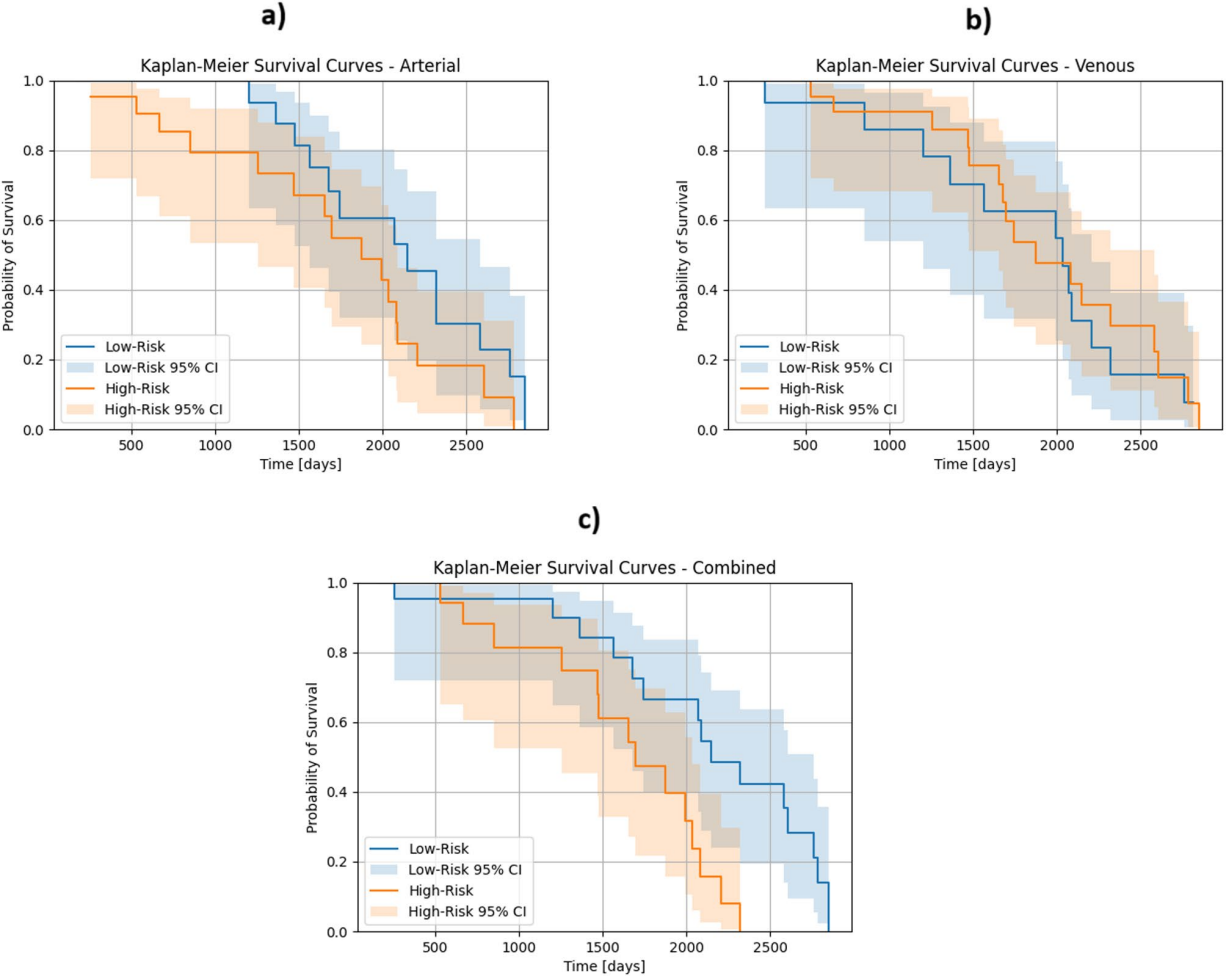
Kaplan-Meier survival probability analysis of patients after pancreatic surgery based on: (**a**) arterial evaluation; (**b**) venous evaluation; and (**c**) combined evaluation. Shaded areas represent 95% confidence intervals for the Kaplan-Meier survival estimates.

Figure 4 depicts the corresponding survival curves. For the arterial (Fig. 4a) and the combined analysis (Fig. 4c), the probability of survival is almost always higher for the patients with a low-risk score than for the ones with a high-risk score, while the survival curves cross multiple times for the venous analysis (Fig. 4b). While survival curves were not statistically significantly different for the arterial (*p* = 0.161) and the venous analysis (*p* = 0.668) alone, a statistically significant difference between the high-risk and the low-risk patients was found for the combined analysis (*p* = 0.007). This is reflected by mostly non-overlapping 95% confidence intervals (shaded areas in Fig. 4c).

The median survival time was 1,693 days for low-risk arterial patients and 1,558.5 days for high-risk arterial patients; 1,564 days for low-risk venous patients and 1,675 days for high-risk venous patients; and 1,743 days for low-risk combined patients and 1,472 days for high-risk combined patients (Table 2). The combined analysis classified 19 patients as high-risk and 23 as low-risk, showing a statistically significant difference in survival between these groups (*p* = 0.007).

These results highlight the improved prognostic accuracy of the combined model compared to the arterial (*p* = 0.161) and venous (*p* = 0.668) analyses alone. Survival rates at predefined postoperative time points (1, 2, 3, and 5 years) vary based on risk group (high vs. low) and model type (Arterial, Venous, and Combined), as shown in Table 2.

Finally, Cox proportional hazards regression analysis was conducted to estimate the predictive ability of risk group classification on survival. The HRs were:

- 1.20 for the arterial model,
- 0.91 for the venous model, and,
- 1.24 for the combined model.

These indicate a higher likelihood of death for high-risk groups in the arterial and combined models, and a lower likelihood in the venous model, although none of the differences reached statistical significance.

### Arterial and combined analyses share their most important features

Table 3 lists the ten most important features per analysis, i.e., the ones with the GBSA model’s highest *feature_importance_* attributes. The features “Shape: sphericity”, “GLSZM: size-zone non-uniformity” and “Shape: surface volume ratio” were the most important features both in the arterial and in the combined analysis, with arterial feature importance scores of 0.26, 0.18 and 0.07 and combined ones of 0.25, 0.12 and 0.06, respectively. “GLSZM: size-zone non-uniformity” with a value of 0.13 was the most important venous feature, followed by “Shape: major axis length” (0.09) and “First order: mean” (0.07). Of note, 7/10 most important combined features were arterial ones.

**Table 3:** Numerical values of key radiomic features in arterial, venous, and combined analyses.

Most important arterial features	Feature importance scores
Shape: sphericity	0.26
Gray level size-zone non-uniformity (GLSZM): size-zone non-uniformity	0.18
Shape: surface volume ratio	0.07
Gray level run length matrix (GLRLM): long run low gray level emphasis	0.05
Shape: maximum three-dimensional (3D) diameter	0.05
GLSZM: gray level non-uniformity	0.03
Shape: maximum two-dimensional (2D) diameter slice	0.02
Gray level co-occurrence matrix (GLCM): cluster shade	0.02
First order: 90th percentile	0.02
First order: 10th percentile	0.02
GLSZM: size-zone non-uniformity	0.13
Shape: major axis length	0.09
First order: mean	0.07
Shape: surface volume ratio	0.06
GLSZM: zone entropy	0.05
Shape: flatness	0.05
Shape: sphericity	0.04
First order: kurtosis	0.03
First order: 90th percentile	0.03
GLCM: cluster shade	0.03
Shape: sphericity (arterial)	0.25
GLSZM: size-zone non-uniformity (arterial)	0.12
Shape: surface volume ratio (arterial)	0.06
First order: total energy (venous)	0.04
Shape: maximum 3D diameter (arterial)	0.04
GLRLM: long run low gray level emphasis (arterial)	0.03
GLCM: inverse difference normalized (venous)	0.03
Shape: major axis length (arterial)	0.02
GLSZM: gray level non-uniformity (arterial)	0.02
GLSZM: size-zone non-uniformity (venous)	0.02

Per analysis, the 10 most important radiomic features and their corresponding feature importance scores are indicated. The features are indicated as “Feature group: feature name”.

## Discussion

This study investigates the potential of radiomic analysis of preoperative contrast-enhanced CT scans for predicting survival outcomes in patients with PaHCa undergoing PPPD, with a focus on the imaging phase after contrast administration. The results emphasize the importance of the integration of venous and arterial phase radiomic features to enhance prognostic performance. As the most important result, our study revealed that individual arterial (*p* = 0.161) and venous (*p* = 0.668) phase features alone were not sufficient to significantly differentiate between high-risk and low-risk patients, while their combination (*p* = 0.007) provided a predictive model. This suggests that the heterogeneous nature of pancreatic tumors could be better modeled by a complete analysis of various imaging phases. The strong predictors identified, such as “Shape: sphericity” and “GLSZM: size-zone non-uniformity,” suggest the possibility of some of the radiomic features to depict the underlying biology and behavior of the tumor, which are responsible for survival prediction.

This additional predictive value of the combined analysis is likely related with the fact that the two contrast phases reflect different aspects of tumor biology. The arterial phase represents the early contrast enhancement, which relates more closely to tumor vascularity and angiogenesis - important features of aggressive pancreatic tumors. The venous phase typically captures late enhancement characteristics and provides information related to overall tissue density, fibrosis, necrosis and stroma. Radiomic features were acquired from both phases, increasing the model’s ability to characterize the tumor’s microenvironment (perfusion and structural heterogeneity) and capture its biological complexity.

Previous studies have established the feasibility of radiomics in pre-operative survival prediction from CT imaging; however, the impact of contrast phase has not yet been analyzed. For instance, Eilaghi et al.^27^ applied radiomics to venous CT images of a biphasic pancreas protocol to predict survival in PDAC patients and showed that the textural features were more significantly correlated with overall survival compared to tumor size. This shows that a radiomics-informed decision can assist in developing a therapeutic plan for patients, helping to identify those with a very poor prognosis who are unlikely to benefit from surgery. Mokhtari et al.^13^ analyzed portal phase CT scans and combined clinical and radiomic features to predict progression-free survival and overall survival in PDAC patients. Their major observation was that the best prediction for overall survival resulted from an integrated approach involving clinical and radiomic features, whereas clinical features provided sufficient predictive powers for the survival of disease progression. Park et al.^14^, using venous phase CT scans, demonstrated that the integration of clinical and radiomic features significantly improved progression-free survival and overall survival predictions. Keyl et al.^15^ also added radiomic parameters to clinical factors for survival prediction, though the CT phase was unspecified (likely venous), demonstrating enhanced prediction accuracy for classifying high-risk and low-risk groups of PDAC patients after surgical resection. Khalvati et al.^17^ conducted a two-institutional radiomic analysis, equally focusing on the venous phase, with the goal to identify single radiomic features that were prognostic for overall survival. Their top three features differ from the ones found in our study, which is likely due to the fact that they considered a different set of radiomic features and delineated only the tumour region. This emphasizes the need for standardized and validated radiomics models. Nevertheless, our study is unique in the sense that we compared models built from venous and arterial radiomic features and their combination, thereby providing a distinct contribution to the existing body of research. It should be noted that an inclusion of arterial features into radiomic models requires a highly standardized contrast injection protocol. This is probably why most studies so far have concentrated on venous images, for which timing requirements during image acquisition are less strict.

The use of GBSA models in this study is noteworthy. GBSA models are known for their ability to handle complex interactions between features and can provide nuanced risk stratification, which is essential in the context of personalized medicine^28,29^. The use of leave-one-out cross-validation^4^ allows for the predictive performance of the models to be robust even with a small cohort and not too optimistic, which is a frequent issue in radiomics studies concerning model overfitting^30,31^. Additionally, the fact that contrast-enhanced CT scans that measure Hounsfield Units could potentially provide more standardized imaging than magnetic resonance imaging, where image contrast can be more varied based on different sequence parameters^32^, is worth mentioning. This standardization could be an additional advantage for CT-based radiomics in clinical practice^33^, although it should be noted that other imaging techniques, e.g. pre-operative diffusion-weighted magnetic resonance imaging, have also been shown capable of predicting survival of patients with PDAC^34^. Apart from survival prediction, radiomic features have been shown to possess potential applications in patients with pancreatic conditions, e.g., resectability prediction or risk of pancreatic fistula^16^.

There are some limitations to this research that need to be considered with care when making conclusions from the findings. Firstly, the comparatively small sample size (*n* = 42) limits the statistical power of our findings and can restrict the generalizability of the model to more heterogeneous patient groups. This raises the risk of overfitting, although we used leave-one-out cross-validation to address this potential problem. Future work should attempt to confirm our findings in larger, multicenter cohorts to maximize the reliability and external applicability of our work. Second, we were limited by a retrospective design that could introduce selection bias, and the patients enrolled may not represent the entire clinical spectrum of PaHCa. To confirm our findings in a broader and more controlled population, it is suggested to conduct prospective studies with standardized imaging and clinical protocols. Third, while the manual segmentation of tumors has been be performed and revised by an expert radiologist, there is still some chance for inter-operator variability^35^. Inter-operator variability can alter the extracted radiomic features, which in turn can lower model performance. As the field progresses, it is expected that semi or fully automated segmentation tools^36^ will be helpful in ensuring reproducibility, reduced operator dependency, and streamline the radiomics workflow in the clinical environment. Although the HRs for high- vs. low-risk groups across all models suggested reduced risk in the low-risk category (Arterial HR: 1.20; Venous HR: 0.91; Combined HR: 1.24), the differences did not reach statistical significance. This may be due to sample size limitations and incomplete event data.

In conclusion, this research presents promising initial evidence for the importance of including both arterial and venous features in the radiomic analysis for predicting survival for PaHCa patients undergoing PPPD. The integration of features from the arterial and venous phase enhanced prognostic accuracy, potentially aiding in personalised treatment planning. Continued research with larger cohorts and advanced segmentation methods will be crucial in establishing radiomics as a reliable tool in the clinical management of PaCa. Finally, our model was not externally validated, which limits its generalizability; future research should incorporate external validation using data from other institutions and scanner types.

## Data Availability

The data generated and analyzed in this study are included in the article. Further details may be provided upon reasonable request to the corresponding author.

## Abbreviations

ASA: American Society of Anesthesiologists
BMI: Body mass index
CT: Computed tomography
GBSA: Gradient boosting survival analysis
GLCM: Gray level co-occurrence matrix
GLDM: Gray level dependence matrix
GLSZM: Gray level size-zone non-uniformity
HR: Hazard ratio
PaCa: Pancreatic cancer
PaHCa: Pancreatic head cancer
PD: Pancreatoduodenectomy
PDAC: Pancreatic ductal adenocarcinoma
PPPD: Pylorus-preserving pancreatoduodenectomy

## References

[1] World Health Organization & International Agency for Research on Cancer. *Global Cancer Observatory: Cancer Today*. https://gco.iarc.fr/today/data/factsheets/cancers/13-Pancreas-fact-sheet.pdf.

[2] Ferlay, J., Partensky, C. & Bray, F. More deaths from pancreatic cancer than breast cancer in the EU by 2017. *Acta Oncol.* **55** (9), 1158–1160 (2016). 27551890 10.1080/0284186X.2016.1197419

[3] Mizrahi, J. D., Surana, R., Valle, J. W. & Shroff, R. T. Pancreatic cancer. *Lancet* **395** (10242), 2008–2020 (2020). 32593337 10.1016/S0140-6736(20)30974-0

[4] Gu, Y. et al. Diagnostic value of combining preoperative inflammatory markers ratios with CA199 for patients with early-stage pancreatic cancer. *BMC Cancer*. **23**, 227 (2023). 36899319 10.1186/s12885-023-10653-4 PMC9999638

[5] Modolell, I., Guarner, L. & Malagelada, J. Vagaries of clinical presentation of pancreatic and biliary tract cancer. *Ann. Oncol.* **4**, 82–84 (1999). 10436792

[6] O’Sullivan, A. W., Heaton, N. & Rela, M. Cancer of the uncinate process of the pancreas: surgical anatomy and clinicopathological features. *Hepatobiliary Pancreat. Dis. Int.* **8** (6), 569–574 (2009). 20007071

[7] Zhang, L., Sanagapalli, S. & Stoita, A. Challenges in diagnosis of pancreatic cancer. *World J. Gastroenterol.* **24** (19), 2047–2060 (2018). 29785074 10.3748/wjg.v24.i19.2047 PMC5960811

[8] Olakowski, M. & Grudzińska, E. Pancreatic head cancer—Current surgery techniques. *Asian J. Surg.* **46** (1), 73–81 (2023). 35680512 10.1016/j.asjsur.2022.05.117

[9] Simoneau, E. et al. Pancreaticoduodenectomy with mesocaval shunt for locally advanced pancreatic adenocarcinoma. *Ann. Surg. Oncol.* **26**, 652 (2019). 30539487 10.1245/s10434-018-07093-x

[10] Gillies, R. J., Kinahan, P. E. & Hricak, H. Radiomics: images are more than pictures, they are data. *Radiology* **278** (2), 563–577 (2016). 26579733 10.1148/radiol.2015151169 PMC4734157

[11] Granata, V. et al. Radiomics-derived data by contrast enhanced magnetic resonance in RAS mutations detection in colorectal liver metastases. *Cancers (Basel)*. **13**, 3 (2021). 10.3390/cancers13030453 PMC7865653 33504085

[12] Barat, M. et al. Artificial intelligence: a critical review of current applications in pancreatic imaging. *Japanese J. Radiol.* **39**, 514–523 (2021). 10.1007/s11604-021-01098-5 33550513

[13] Mokhtari, A. et al. Development of clinical radiomics-based models to predict survival outcome in pancreatic ductal adenocarcinoma: a multicenter retrospective study. *Diagnostics* **14** (7), 712 (2024). 38611625 10.3390/diagnostics14070712 PMC11011556

[14] Ark, S. et al. CT radiomics–based preoperative survival prediction in patients with pancreatic ductal adenocarcinoma. *Am. J. Roentgenol.* **217** (5), 1104–1112 (2021). 34467768 10.2214/AJR.20.23490

[15] Keyl, J. et al. Multimodal survival prediction in advanced pancreatic cancer using machine learning. *ESMO Open.* **7** (5), 100555 (2022). 35988455 10.1016/j.esmoop.2022.100555 PMC9588888

[16] Pacella, G. et al. Pancreatic ductal adenocarcinoma: update of CT-based radiomics applications in the pre-surgical prediction of the risk of post-operative fistula, resectability status and prognosis. *Resectability Status Prognosis*. **12** (23), 7380 (2023). 10.3390/jcm12237380 PMC10707069 38068432

[17] Khalvati, F. et al. Prognostic value of CT radiomic features in resectable pancreatic ductal adenocarcinoma. *Sci. Rep.* **9** (1), 5449 (2019). 30931954 10.1038/s41598-019-41728-7 PMC6443807

[18] Saklad, M. Grading of patients for surgical procedures. *Anesthesiology* **2** (3), 281–284 (1941).

[19] Yushkevich, P. A. User-guided 3D active contour segmentation of anatomical structures: significantly improved efficiency and reliability. *Neuroimage* **31**, 1116–1128 (2006). 16545965 10.1016/j.neuroimage.2006.01.015

[20] van Griethuysen, J. et al. Computational radiomics system to Decode the radiographic phenotype. *Cancer Res.* **77**, 21 (2017). 10.1158/0008-5472.CAN-17-0339 PMC5672828 29092951

[21] *Radiomic Features*. https://pyradiomics.readthedocs.io/en/latest/features.html#radiomics.gldm.RadiomicsGLDM.

[22] Pölsterl, S. scikit-survival: A library for time-to-event analysis built on top of scikit-learn. *J. Mach. Learn. Res.* **21**, 1–6 (2020).34305477

[23] Pedregosa, F. et al. Machine learning in Python. *J. Mach. Learn. Res.* **12**, 2825–2830 (2011).

[24] Pölsterl, S. *Scikit-Survival: A Library for Time-to-Event Analysis Built on Top of Scikit-Learn*. https://scikit-survival.readthedocs.io/en/stable/api/generated/sksurv.ensemble.GradientBoostingSurvivalAnalysis.html (2020).

[25] Barraclough, H., Simms, L. & Govindan, R. Biostatistics primer: what a clinician ought to know: hazard ratios. *J. Thorac. Oncol.* **6** (6), 978–982 (2011). 21623277 10.1097/JTO.0b013e31821b10ab

[26] Akoglu, H. User’s guide to correlation coefficients. *Turk. J. Emerg. Med.* **18** (3), 91–93 (2018). 30191186 10.1016/j.tjem.2018.08.001 PMC6107969

[27] Eilaghi, A. et al. CT texture features are associated with overall survival in pancreatic ductal adenocarcinoma—a quantitative analysis. *BMC Med. Imaging*. **17**, 38. 10.1186/s12880-017-0209-5 (2017). 28629416 10.1186/s12880-017-0209-5 PMC5477257

[28] Vasilev, I., Petrovskiy, M. & Mashechkin, I. Sensitivity of survival analysis metrics. *Mathematics* **11** (20), 4246 (2023).

[29] Peng, Z. H. et al. Development of machine learning prognostic models for overall survival of prostate cancer patients with lymph node-positive. *Sci. Rep.* **13**, 18424. 10.1038/s41598-023-45804-x (2023). 37891423 10.1038/s41598-023-45804-x PMC10611782

[30] Bradshaw, T. J., Huemann, Z., Hu, J. & Rahmim, A. A guide to cross-validation for artificial intelligence in medical imaging. *Radiol. Artif. Intell.* **5**, e220232. 10.1148/ryai.220232 (2023). 10.1148/ryai.220232 PMC10388213 37529208

[31] Wang, H. et al. Contrast-enhanced computed tomography radiomics in predicting primary site response to neoadjuvant chemotherapy in high-risk neuroblastoma. *Abdom. Radiol.* **48**, 976–986. 10.1007/s00261-022-03774-0 (2023). 10.1007/s00261-022-03774-0 36571609

[32] Florkow, M. C. & Et Magnetic resonance imaging versus computed tomography for three-dimensional bone imaging of musculoskeletal pathologies: a review. *J. Magn. Reson. Imaging*. **56** (1), 11–34. 10.1002/jmri.28067 (2022). 35044717 10.1002/jmri.28067 PMC9305220

[33] Zhou, C. et al. CT-based radiomics nomogram May predict who can benefit from adaptive radiotherapy in patients with locally advanced NSCLC. *Radiother. Oncol.* **183**, 109637. 10.1016/j.radonc.2023.109637 (2023). 36963440 10.1016/j.radonc.2023.109637

[34] Kaissis, G. et al. A machine learning model for the prediction of survival and tumor subtype in pancreatic ductal adenocarcinoma from preoperative diffusion-weighted imaging. *Eur. Radiol. Exp.* **3** (1), 1–9 (2019). 31624935 10.1186/s41747-019-0119-0 PMC6797674

[35] Seo, K. et al. Semantic segmentation of pancreatic cancer in endoscopic ultrasound images using deep learning approach. *Cancers* **14** (20), 5111 (2022). 36291895 10.3390/cancers14205111 PMC9600976

[36] Azimbagirad, M. et al. Robust semi-automatic segmentation method: An expert assistant tool for muscles in CT and MR data. *Comput. Methods Biomech. Biomed. Eng. Imaging Vis.* **11**, 7 (2024).

